# Supplementary figures and images for: Kilohertz waveforms optimized to produce closed-state Na+ channel inactivation eliminate onset response in nerve conduction block
Source: PLoS Comput Biol. 2020 Jun 15;16(6):e1007766. doi: 10.1371/journal.pcbi.1007766 (PMC7316353; doi:10.1371/journal.pcbi.1007766)

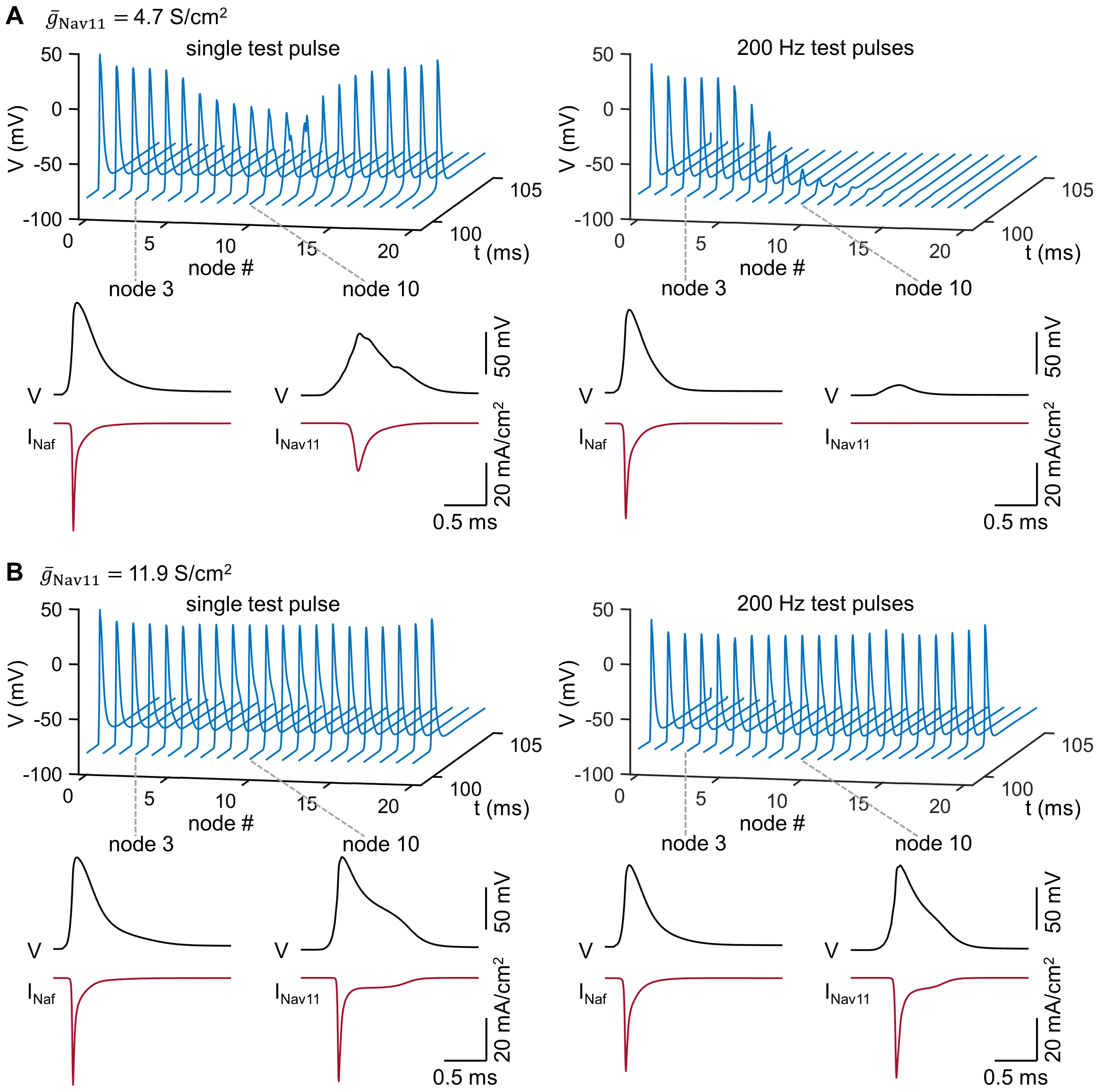

Supplement: S1 Fig — Propagation of a test AP initiated in node 0 along the 21-node and 10 μm model at (A) g¯Nav11 = 4.7 S/cm2 and (B) 11.9 S/cm2. g¯Nav11 = 4.7 S/cm2 was the minimum conductance for faithfully propagating a single AP along the axon, and g¯Nav11 = 11.9 S/cm2 was the minimum conductance for faithfully propagating spike trains at rates of up to 400 Hz along the axon. INaf was recorded in node 3, and INav11 was recorded in node 10. Left panels: a single test pulse (width: 0.1 ms and amplitude: 2.5Ith) was delivered at the node 0. Right panels: 200 Hz test pulses were delivered at the node 0. Maximum conductance of HH-type INaf was g¯Naf = 3.0 S/cm2, maximum conductance of HH-type INap was g¯Nap = 0.01 S/cm2, and maximum conductance of Markov-type INav16 was g¯Nav16 = 0.01 S/cm2. (TIF) [file pcbi.1007766.s001.tif]

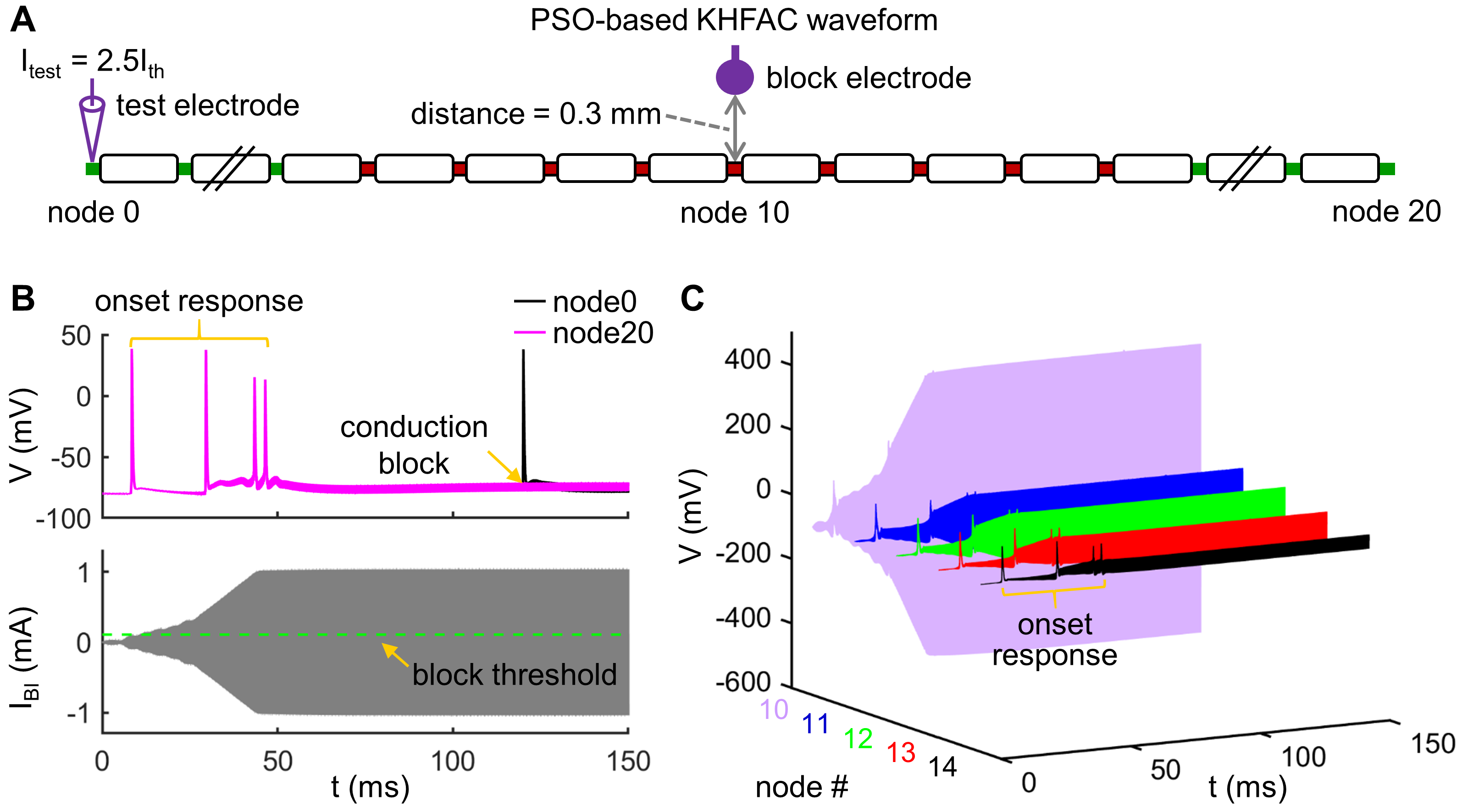

Supplement: S2 Fig — (A) Simulation setup. A monopolar block electrode was placed 0.3 mm over the central node of 21-node 10 μm diameter model nerve fiber. An intracellular test pulse (width: 0.1 ms and amplitude: 2.5Ith) was delivered at node 0 to generate a propagating AP at t = 120 ms. (B) Transmembrane voltages (top) recorded in node 0 and node 20 in response to KHFAC waveform IBI (bottom). A scale factor of 1.5 was used to design the envelope of IBI, and the green dotted line was the block threshold at electrode-fiber distance of 0.3 mm. (C) Voltage responses recorded in node 10 to node 14. (TIF) [file pcbi.1007766.s002.TIF]

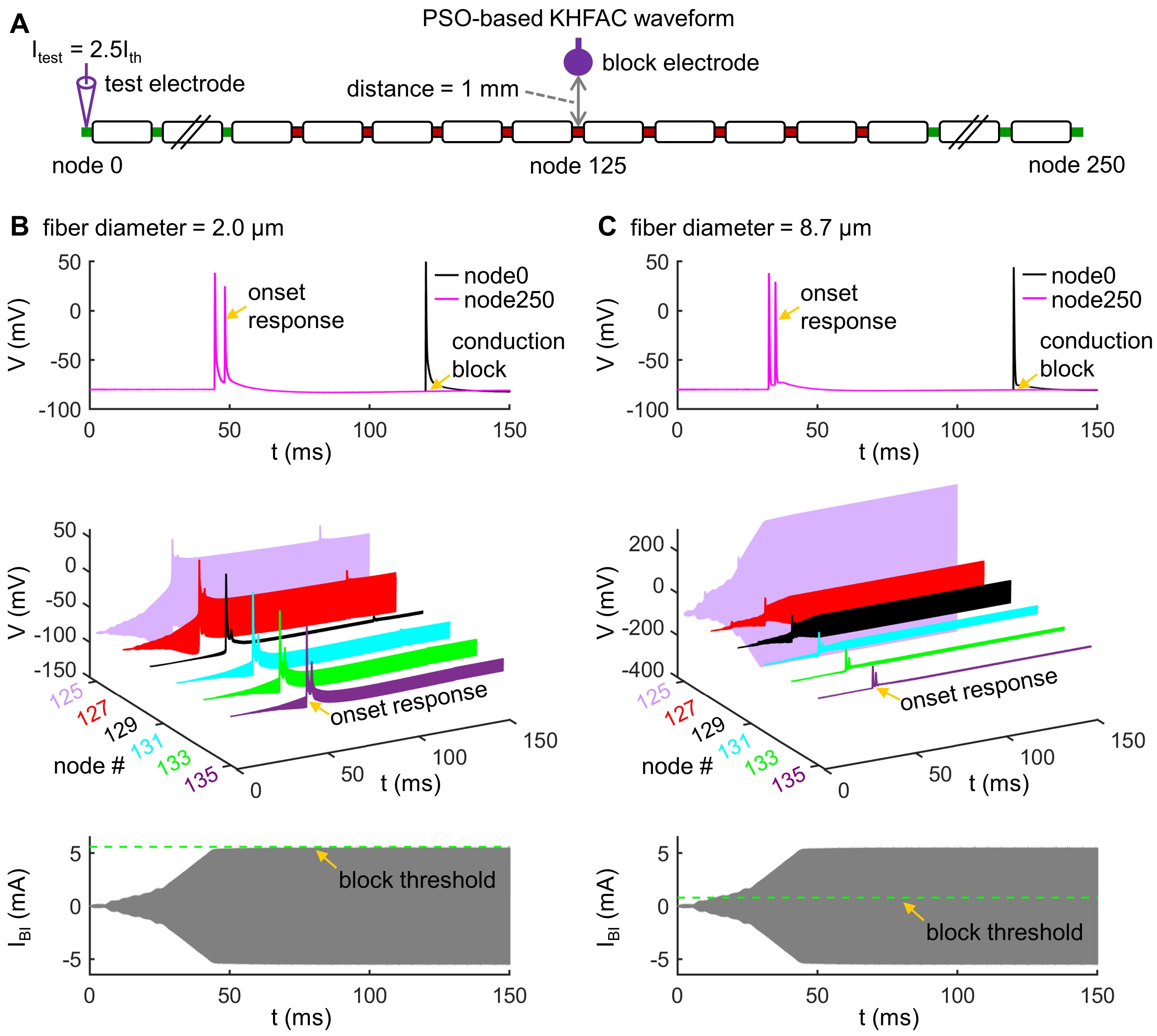

Supplement: S3 Fig — (A) Simulation setup. A monopolar block electrode was placed 1 mm over node 125 and delivered KHFAC waveform IBI to a 251-node model. An intracellular test pulse (width: 0.1 ms and amplitude: 2.5Ith) was delivered at node 0 to generate a propagating AP at t = 120 ms. Markov-type Nav 1.1 and Nav 1.6 channels were implemented in node 121 to node 129. (B) Block of a test AP along the axon by IBI with a fiber diameter of 2.0 μm. (C) Block of a test AP along the axon by IBI with a fiber diameter of 8.7 μm. In (B) and (C), the green dotted lines were the block threshold at each fiber diameter. (TIF) [file pcbi.1007766.s003.TIF]

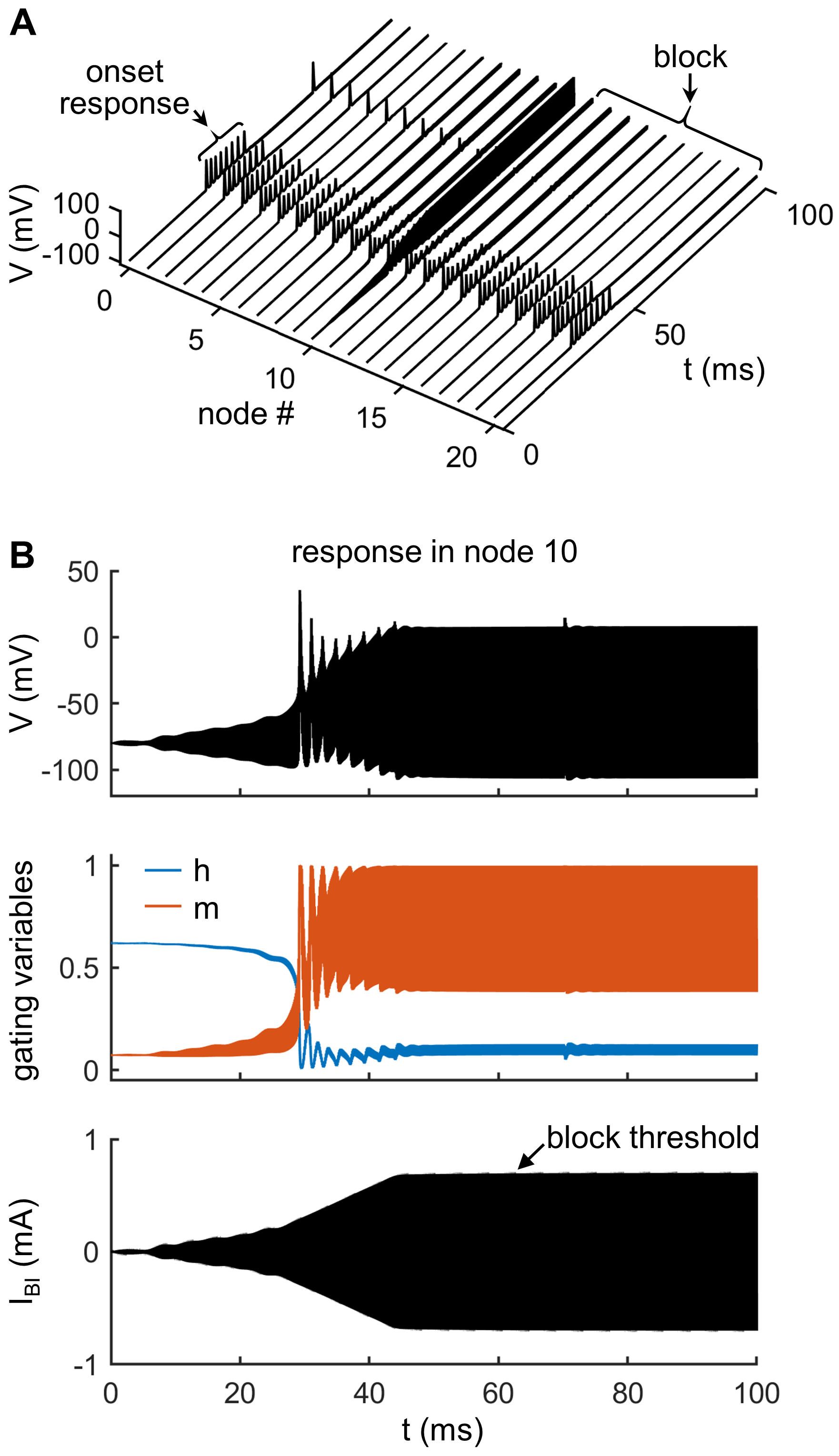

Supplement: S4 Fig — (A) Block of a test AP along the 21-node 10 μm diameter model nerve fiber by PSO-based waveform IBI. No Markov-type VGSCs were implemented in the central nodes. The block electrode was placed 1 mm above node 10, and a single test pulse (width: 0.1 ms and amplitude: 2.5Ith) was delivered at node 0 to generate a propagating AP at t = 70 ms. (B) Top panel: transmembrane voltage recorded in node 10 in response to KHFAC waveform IBI. Center panel: activation gating variable m and inactivation gating variable h of fast Na+ current. Bottom panel: PSO-based KHFAC waveform IBI with a plateau amplitude scaled to the block threshold. (TIF) [file pcbi.1007766.s004.TIF]

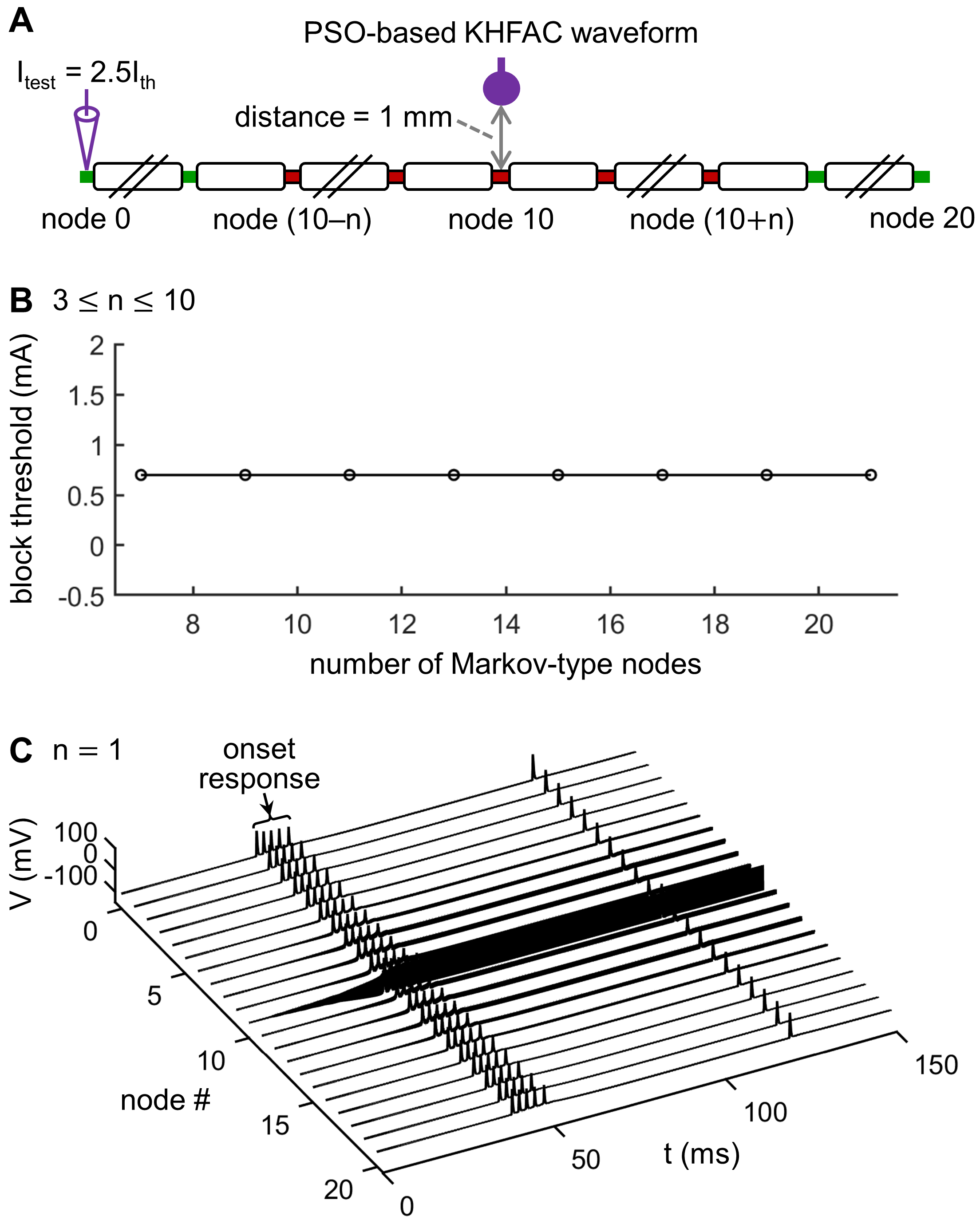

Supplement: S5 Fig — (A) Simulation setup. We implemented Nav 1.1 and Nav 1.6 channels in node (10−n) to node (10+n), where 0 ≤ n ≤ 10. A monopolar block electrode was placed 1 mm over node 10, and an intracellular test pulse (width: 0.1 ms and amplitude: 2.5Ith) was delivered at node 0 to generate a propagating AP at t = 120 ms. (B) Block threshold as a function of the number of Markov-type nodes. At 3 ≤ n ≤ 10, PSO-based KHFAC waveform IBI produced onset-free conduction block. At 0 ≤ n ≤ 2, no conduction block occurred and onset firing was activated by IBI. (C) Propagation of a test AP along the axon by IBI (n = 1). A scale factor of 1.0 was used to design the envelope of IBI, and the number of Markov-type nodes was three. (TIF) [file pcbi.1007766.s005.TIF]

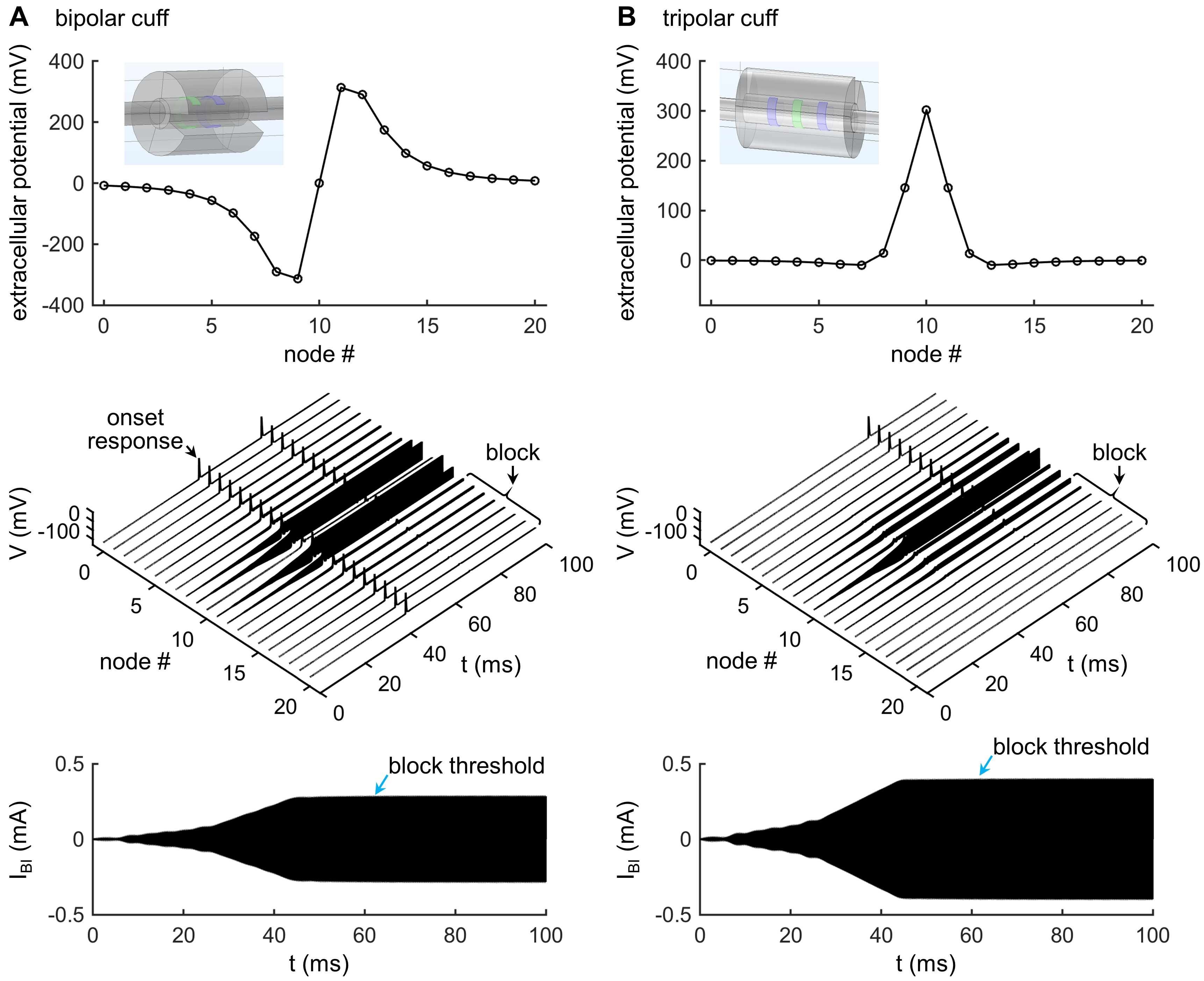

Supplement: S6 Fig — (A) We interpolated the extracellular potential at each segment of 21-node hybrid model from a finite element model (FEM) of the rat tibial nerve, which had a bipolar nerve cuff and a model nerve fiber that was 0.398 mm away from the electrode. Since the bipolar cuff resulted in the largest depolarization in node 11, we applied PSO algorithm to generate a DC waveform IPSO to drive the optimized voltage trajectory VPSO in node 11. The envelop of KHFAC waveform IBI was determined by multiplying IPSO by a scale factor of 0.73, and the plateau amplitude of IBI was the block threshold. The resulting KHFAC waveform blocked nerve conduction with activation of an onset response. (B) We used a FEM of a tripolar cuff on the rat tibial nerve to interpolate the extracellular potentials of 21-node hybrid model, and the electrode-fiber distance was 0.398 mm. The DC waveform IPSO was designed to drive VPSO in node 10. A scale factor of 0.93 was applied to scale the plateau amplitude of IBI to the block threshold. The resulting KHFAC waveform produced onset-free conduction block. A single test pulse (width: 0.1 ms and amplitude: 2.5Ith) was injected in node 0 at t = 70 ms. Fiber diameter was 10 μm. (TIF) [file pcbi.1007766.s006.TIF]

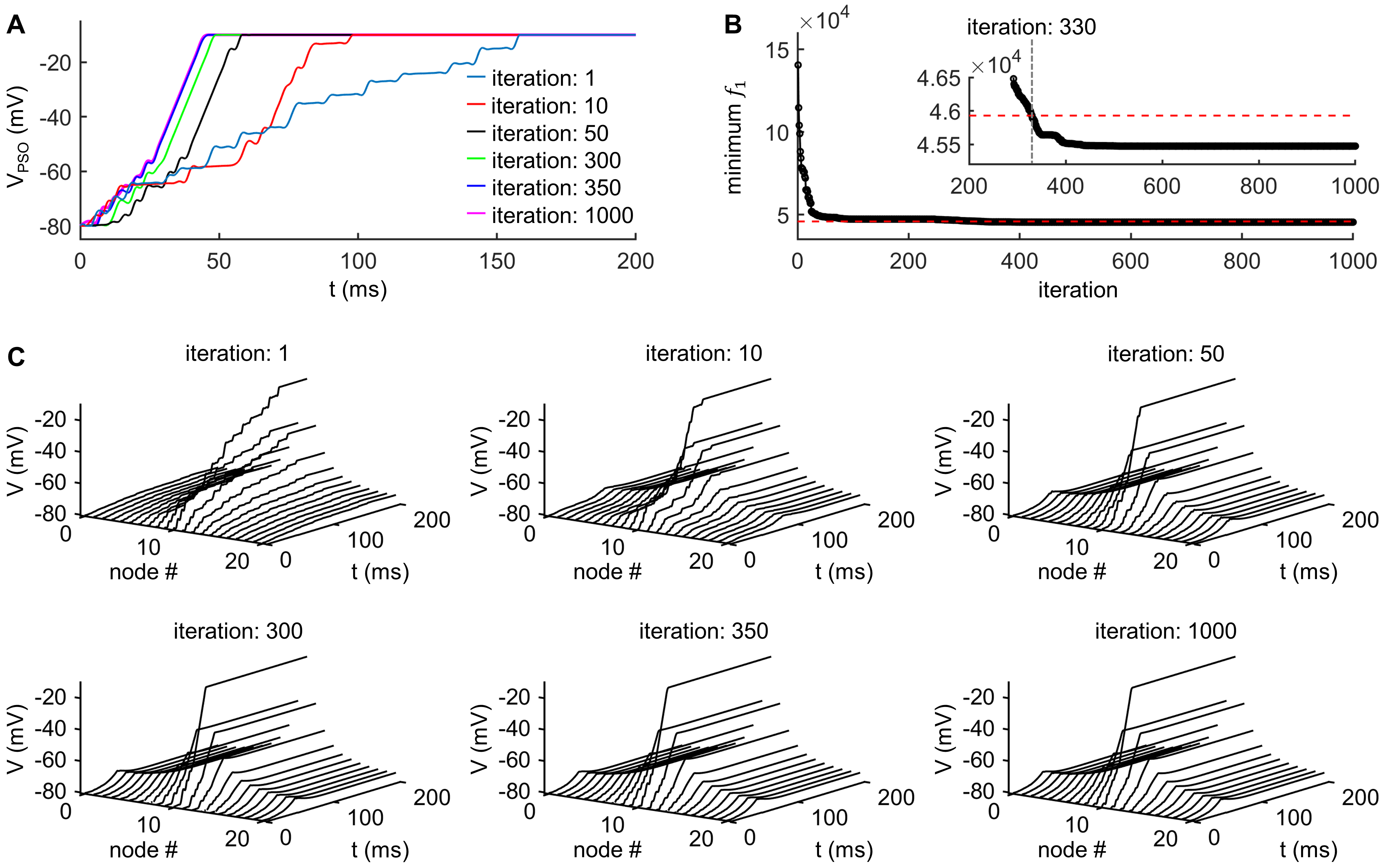

Supplement: S7 Fig — (A) Changes of transmembrane voltage trajectory across iterations. The sequence of plots show the voltage profile with the minimum cost f1 at each indicated iteration. (B) Minimum cost f1 of 50 particles at each iteration. Red dotted line is 1.01 times the minimum cost at the final generation. (C) Voltage response recorded in each node when applying the voltage profile at each indicated iteration as a series of voltage clamps at node 10. Fiber diameter was 10 μm. (TIF) [file pcbi.1007766.s007.TIF]

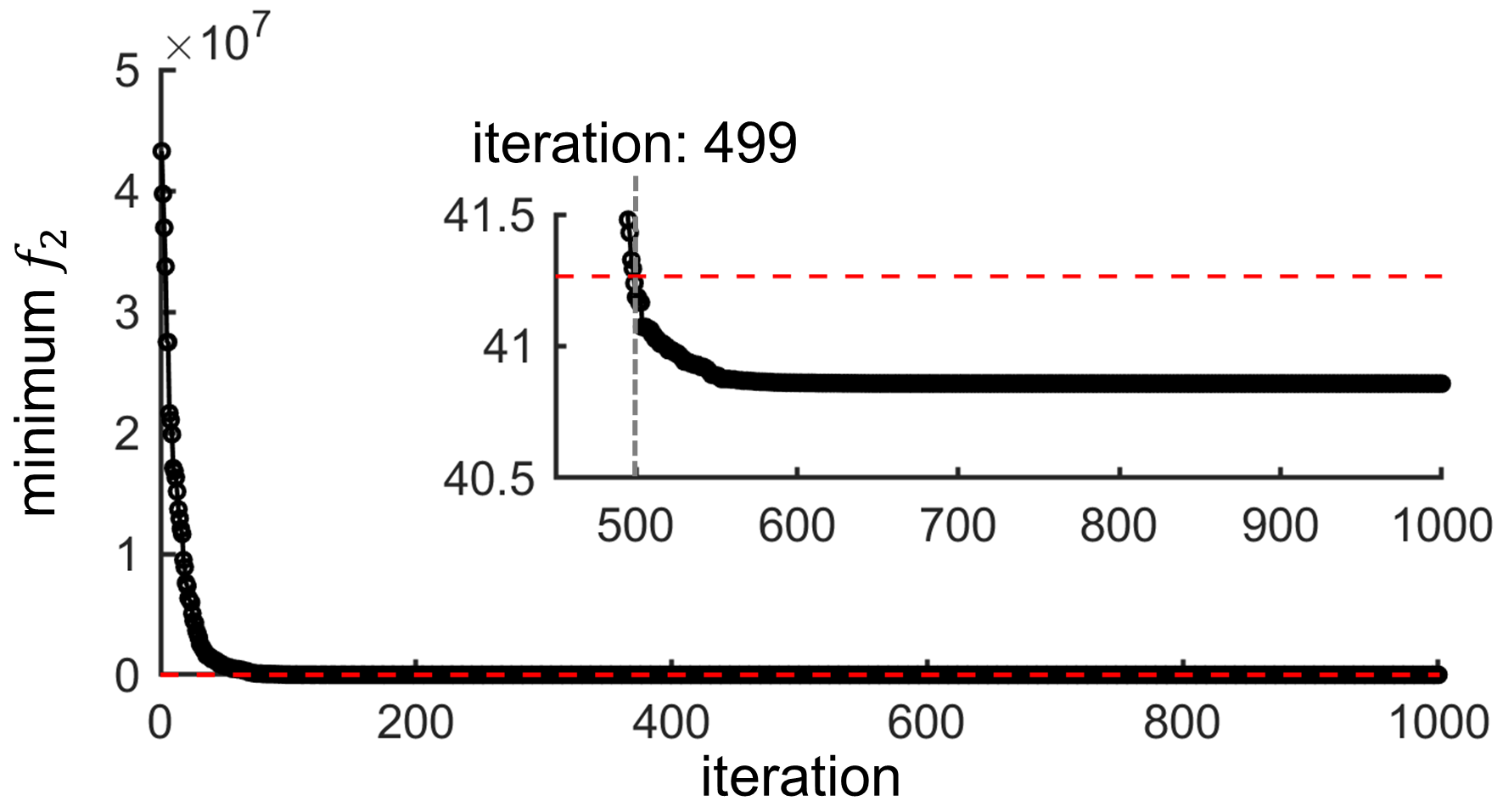

Supplement: S8 Fig — Red dotted line was 1.01 times the minimum cost at the final generation. (TIF) [file pcbi.1007766.s008.TIF]
