# Supplementary material for: Kilohertz waveforms optimized to produce closed-state Na+ channel inactivation eliminate onset response in nerve conduction block
Source: PLoS Comput Biol. 2020 Jun 15;16(6):e1007766. doi: 10.1371/journal.pcbi.1007766 (PMC7316353; doi:10.1371/journal.pcbi.1007766)
Supplement: S1 Table — (DOCX) [file pcbi.1007766.s009.docx]

**S1 Table.** **Geometric parameters of MRG models**

| fiber diameter | 2.0 μm [1] | 8.7 μm [2] | 10.0 μm [2] |
| --- | --- | --- | --- |
| internodal length | 200 μm | 1000 μm | 1150 μm |
| number of myelin lemella | 30 | 110 | 120 |
| node length | 1 μm | 1 μm | 1 μm |
| node diameter | 1.4 μm | 2.8 μm | 3.3 μm |
| MYSA length | 3 μm | 3 μm | 3 μm |
| MYSA diameter | 1.4 μm | 2.8 μm | 3.3 μm |
| MYSA periaxonal space width | 0.002 μm | 0.002 μm | 0.002 μm |
| FLUT length | 10 μm | 40 μm | 46 μm |
| FLUT diameter | 1.6 μm | 5.8 μm | 6.9 μm |
| FLUT periaxonal space width | 0.004 μm | 0.004 μm | 0.004 μm |
| STIN length | 57.7 μm | 152.2 μm | 175.2 μm |
| STIN diameter | 1.6 μm | 5.8 μm | 6.9 μm |
| STIN periaxonal space width | 0.004 μm | 0.004 μm | 0.004 μm |

**References**

1. McIntyre CC, Grill WM, Sherman DL, Thakor NV. Cellular effects of deep brain stimulation: model-based analysis of activation and inhibition. J Neurophysiol. 2004 Apr; 91(4): 1457–69. doi: 10.1152/jn.00989.2003
2. McIntyre CC, Richardson AG, Grill WM. Modeling the excitability of mammalian nerve fibers: influence of afterpotentials on the recovery cycle. J Neurophysiol. 2002 Feb; 87(2): 995–1006. doi: 10.1152/jn.00353.2001
