# Supplementary material for: Kilohertz waveforms optimized to produce closed-state Na+ channel inactivation eliminate onset response in nerve conduction block
Source: PLoS Comput Biol. 2020 Jun 15;16(6):e1007766. doi: 10.1371/journal.pcbi.1007766 (PMC7316353; doi:10.1371/journal.pcbi.1007766)
Supplement: S2 Table — (DOCX) [file pcbi.1007766.s010.docx]

**S2 Table.** **Electrical parameters of MRG models**

| parameters | value [1] |
| --- | --- |
| maximum fast Na^+^ conductance $\bar{g}_{\mathrm{Naf}}$ | 3.0 S/cm^2^ |
| maximum slow K^+^ conductance $\bar{g}_{\mathrm{KS}}$ | 0.08 S/cm^2^ |
| maximum persistent Na^+^ conductance $\bar{g}_{\mathrm{Nap}}$ | 0.01 S/cm^2^ |
| nodal leakage conductance $g_{L}$ | 0.007 S/cm^2^ |
| Na^+^ reversal potential *E*_Na_ | 50 mV |
| K^+^ reversal potential *E*_K_ | $-$90 mV |
| leakage reversal potential *E*_L_ | $-$90 mV |
| nodal capacitance *C*_n_ | 2 μF/cm^2^ |
| internodal capacitance *C*_i_ | 2 μF/cm^2^ |
| myelin capacitance *C*_m_ | 0.1 μF/cm^2^ |
| myelin conductance *G*_m_ | 0.001 S/cm^2^ |
| MYSA conductance *g*_a_ | 0.001 S/cm^2^ |
| FLUT conductance *g*_f_ | 0.0001 S/cm^2^ |
| STIN conductance *g*_i_ | 0.0001 S/cm^2^ |
| axoplasmic resistivity *ρ*_a_ | 70 $\Omega\cdot cm$ |
| periaxonal resistivity *ρ*_p_ | 70 $\Omega\cdot cm$ |

**Reference**

1. McIntyre CC, Richardson AG, Grill WM. Modeling the excitability of mammalian nerve fibers: influence of afterpotentials on the recovery cycle. J Neurophysiol. 2002 Feb; 87(2): 995–1006. doi: 10.1152/jn.00353.2001
