# Supplementary material for: Kilohertz waveforms optimized to produce closed-state Na+ channel inactivation eliminate onset response in nerve conduction block
Source: PLoS Comput Biol. 2020 Jun 15;16(6):e1007766. doi: 10.1371/journal.pcbi.1007766 (PMC7316353; doi:10.1371/journal.pcbi.1007766)
Supplement: S3 Table — (DOCX) [file pcbi.1007766.s011.docx]

**S3 Table.** Parameters of the transition rates for Na_v_ 1.1 and Na_v_ 1.6 channels [1]

|  | $B_{hyp}$ | $V_{hyp}$ | $k_{hyp}$ | $B_{dep}$ | $V_{dep}$ | $k_{dep}$ |
| --- | --- | --- | --- | --- | --- | --- |
| Nav 1.1 channels | | | | | | |
| C1C2 | 0 | 0 | 0 | 18 | $-$7 | $-$10 |
| C2C1 | 3 | $-$37 | 10 | 18 | $-$7 | $-$10 |
| C2O1 | 0 | 0 | 0 | 18 | $-$7 | $-$10 |
| O1C2 | 3 | $-$37 | 10 | 18 | $-$7 | $-$10 |
| C2O2 | 0 | 0 | 0 | 0.08 | $-$10 | $-$15 |
| O2C2 | 2 | $-$50 | 7 | 0.2 | $-$20 | $-$10 |
| O1I1 | 8 | $-$37 | 13 | 17 | $-$7 | $-$15 |
| I1O1 | 0.00001 | $-$37 | 10 | 0 | 0 | 0 |
| I1C1 | 0.21 | $-$61 | 7 | 0 | 0 | 0 |
| C1I1 | 0 | 0 | 0 | 0.3 | $-$61 | $-$5.5 |
| I1I2 | 0 | 0 | 0 | 0.0015 | $-$90 | $-$5 |
| I2I1 | 0.0075 | $-$90 | 15 | 0 | 0 | 0 |
| Nav 1.6 channels | | | | | | |
| C1C2 | 0 | 0 | 0 | 14 | $-$8 | $-$10 |
| C2C1 | 2 | $-$38 | 9 | 14 | $-$8 | 10 |
| C2O1 | 0 | 0 | 0 | 14 | $-$18 | $-$10 |
| O1C2 | 4 | $-$48 | 9 | 14 | $-$18 | $-$10 |
| C2O2 | 0 | 0 | 0 | 0.0001 | $-$10 | $-$8 |
| O2C2 | 0.0001 | $-$55 | 10 | 0.0001 | $-$20 | $-$5 |
| O1I1 | 6 | $-$40 | 13 | 10 | 15 | $-$18 |
| I1O1 | 0.00001 | $-$40 | 10 | 0 | 0 | 0 |
| I1C1 | 0.1 | $-$86 | 9 | 0 | 0 | 0 |
| C1I1 | 0 | 0 | 0 | 0.08 | $-$55 | $-$12 |
| I1I2 | 0 | 0 | 0 | 0.00022 | $-$50 | $-$5 |
| I2I1 | 0.0018 | $-$90 | 30 | 0 | 0 | 0 |

**Reference**

1. Balbi P, Massobrio P, Hellgren Kotaleski J. A single Markov-type kinetic model accounting for the macroscopic currents of all human voltage-gated sodium channel isoforms. PLoS Comput Biol. 2017 Sep 1; 13(9): e1005737. doi: 10.1371/journal.pcbi.1005737
