# Supplementary material for: Kilohertz waveforms optimized to produce closed-state Na+ channel inactivation eliminate onset response in nerve conduction block
Source: PLoS Comput Biol. 2020 Jun 15;16(6):e1007766. doi: 10.1371/journal.pcbi.1007766 (PMC7316353; doi:10.1371/journal.pcbi.1007766)
Supplement: Attachment — Submitted filename: Comments V2.pdf [file pcbi.1007766.s012.pdf]

Kilohertz waveforms optimized to produce closed-state Na+ channel inactivation eliminate onset response in nerve conduction block

PLOS Computational Biology.

Abstract/Summary

- In previous papers, KHFAC is used instead of KHF
- Mention autonomic applications in both the abstract and summary

Introduction

- Has CSI been validated in different cell types? Several of the references are for cardiac cells. Include a list of what types of cells it has been observed in.
- Mention that the Markov model is validated across electrophysiological data from all VGSC isoforms (Balbi article)
- There are a lot of different optimization techniques, why did you chose PSO?

Results

- There are a couple of things that are fully explained in the methods, but could use a little bit more explanation here
    - State why were HH channels unable to represent the dependence of inactivation on activation.
    - Could use a little more background on the PSO algorithm before launching into the way you are using it. Mention why it was selected.
    - "We defined a population of 50 particles as the membrane voltages used to control a series of voltage clamps at node 10." Clarify that the membrane voltage is a voltage profile.
- These items don't seem to be well explained in the methods either
    - Why only in the middle nodes?
    - Why change the conductance densities? Is there a reference for this?

KHFAC waveform

- Describe why you thought this would work. KHFAC generates a dynamic depolarization which should be able to drive the membrane voltage?
- What is the purpose of the 1.5 scale factor? If you have created an optimized waveform, why are you changing it?

Discussion

- I don't think that applying a waveform that has been optimized to one model to another model produces a valid comparison. I not sure what you are trying to demonstrate by altering the number of Markov-type nodes? It isn't clear at this point why you only changed a select number of nodes.

- In reference to the Gerges paper: "The amplitude transition in their KHF waveform was opposite with our PSO based waveform" This may be true, but they are also changing the frequency. Comparing your results with the Miles paper is a more appropriate comparison.
- This is basically an KHFAC amplitude ramping waveform that has been tested in vivo. Spend more time talking about the Miles paper and include this reference as well

  Vrabec, T.L., et al., Reduction of the onset response in kilohertz frequency alternating current nerve block with amplitude ramps from non-zero amplitudes. Journal of neuroengineering and rehabilitation, 2019. **16**(1): p. 80-80.

- The Franke paper included high capacitance materials as well as a charge balanced waveform.
- Mention the ramping papers again in the paragraph about the bipolar electrodes. The ramping papers both used bipolars and are a direct comparison to what you are doing.

Methods

- Implementation of Markov type VGSCs
  - There still isn't any reason for only doing nodes 6-14
  - It looks like the reason that you needed to change the conductance was because of the transition between HH and Markov channels which begs the question of why all the nodes aren't Markov?
- Particle Swarm Optimization
  - Why was this optimization approach chosen? (or include this in the results section)
