# Supplementary material for: Kilohertz waveforms optimized to produce closed-state Na+ channel inactivation eliminate onset response in nerve conduction block
Source: PLoS Comput Biol. 2020 Jun 15;16(6):e1007766. doi: 10.1371/journal.pcbi.1007766 (PMC7316353; doi:10.1371/journal.pcbi.1007766)
Supplement: Attachment — Submitted filename: PCOMPBIOL-D-19-02003_Response.docx [file pcbi.1007766.s013.docx]

PCOMPBIOL-D-19-02003

“Kilohertz waveforms optimized to produce closed-state Na^+^ channel inactivation eliminate onset response in nerve conduction block”

Guosheng Yi and Warren M. Grill

**RESPONSE TO REVIEWERS**

**Reviewer #1**

An interesting, drug-free method for blocking nerve conduction relies on inactivation of voltage-gated sodium (Nav) channels via externally applied, high-frequency electrical stimuli. This technique can potentially be used to treat pain and spasticity. However, one caveat is that the nerve responds to the onset of the stimulus, which can cause transient muscle contractions and pain.

Yi and Grill propose here a procedure for optimizing the stimulus waveform in such a way that the onset response is eliminated. To generate action potentials, Nav channels first activate, open and generate current, then inactivate and remain inactivated for a period of time that makes the nerve refractory to stimuli. However, Nav channels can also inactivate without opening, a process termed closed state inactivation (CSI). The kHz waveform designed by Yi and Grill exploits CSI.

The problem addressed is important and interesting, the modeling work is good, and the manuscript is clear and well written. Overall, I really enjoyed reading the manuscript and I didn't see any major issue. I only have a number of minor comments and suggestions.

1. Fig. 2: I think it might be good to show what happens when a standard voltage step is applied, for readers who are less familiar with Nav channels, and also to put the magnitude of the residual INa in context. Otherwise, the current graph in A is uninterpretable.

Response: We agree with the Reviewer and added several panels in Fig. 2 to show the responses in Na_v_ 1.1 and Na_v_ 1.6 channels when a standard voltage step (from –80 mV to –10 mV) was applied to the central node as a voltage clamp (for convenience, included herein as Fig. R1A-R1C). At the resting potential of –80 mV, the two voltage-gated sodium channels (VGSCs) were in closed states C1. After the onset of the voltage step to –10 mV, both Na_v_ 1.1 and Na_v_ 1.6 channels first entered open states O1 and then inactivated (Fig. R1B), i.e., open-state inactivation (OSI) occurred. The magnitude of *I*_Nav11_ and *I*_Nav16_ respectively reached –81.85 mA/cm^2^ and –0.10 mA/cm^2^. The conductance density of Na_v_ 1.6 channels (i.e., 0.01 mS/cm^2^) was much smaller than that of Na_v_ 1.1 channels (i.e., 11.9 mS/cm^2^), and *I*_Nav11_ was much higher than *I*_Nav16_. There was an action potential that propagated in the hybrid model axon (Fig. R1C) at the onset of voltage step. We revised Fig. 2 and included these additional simulations in the second paragraph of the first subsection of the Results in the revised manuscript.

**Fig. R1. Trajectory of transmembrane voltage to cause Na^+^ CSI.** **(A)** Top panel: Voltage step profile *V*_step_. The membrane voltage was increased from the resting potential of –80 mV to –10 mV at 25 ms. Center panel: Na_v_ 1.1 current during *V*_step_. Bottom: Na_v_ 1.6 current during *V*_step_ **(B)** Top panel: fractions of Na_v_ 1.1 channels in states C1 (C11), I1 (I11), I2 (I12), and O1 (O11) during *V*_step_. Bottom panel: fractions of Na_v_ 1.6 channels in states C1 (C61), I1 (I61), I2 (I62), and O1 (O61) during *V*_step_. **(C)** Voltage response recorded in each node when applying *V*_step_ at node 10. **(D)** Top panel: PSO-generated optimized voltage profile *V*_PSO_. Center panel: Na_v_ 1.1 current during *V*_PSO_. Bottom panel: Na_v_ 1.6 current during *V*_PSO_. **(E)** Top panel: C11, I11, I12, and O11 of Na_v_ 1.1 channels during *V*_PSO_. Bottom panel: C61, I61, I62, and O61 of Na_v_ 1.6 channels during *V*_PSO_. **(F)** Voltage response recorded in each node when applying *V*_PSO_ as a series of voltage clamps at node 10.

In contrast to the voltage clamp step, there was no action potential generated in response to the optimized voltage profile, *V*_PSO_. After depolarization with *V*_PSO_, the fraction of either Na_v_ 1.1 and Na_v_ 1.6 channels in C1 was gradually reduced, the fractions in I2 increased, and there was little increase in the fraction in O1. Thus, more Na_v_ 1.1 and Na_v_ 1.6 channels were driven to inactivation directly from the resting state, which reduced the availability of each channel to conduct and participate in AP generation. We revised the text in the fourth paragraph of the first subsection in Results to make these differences clear.

2. The states could be directly called C11, I11, etc., instead of SC11, SI11, etc.

Response: We agree with the Reviewer and revised the terminology to C11, I11, etc.

3. Do I understand correctly that the voltage was clamped only at one node (10), and the rest of the axon was completely free? If so, I am still a little surprised that no APs were generated (escaped) farther away from node 10. I think it's really important to show a waveform that is not fully optimized and permits APs to escape, side by side with the optimized waveform, so readers understand what has been achieved here. The authors might want to cite a paper where this phenomenon of escaped APs is actually exploited to selectively silence axonal Nav channels (Milescu at al., 2010).

Response: Yes, the voltage clamp of the optimized voltage profile, *V*_PSO_, was applied only at the central node and the rest of the axon was completely free. As shown in Fig. R1, above, the voltage clamp step did result in a propagation action potential, while *V*_PSO_ did not. We revised Fig. 2 to enable the side by side comparison suggested by the Reviewer.

Milescu *et al.* [1] isolated the somatic Na^+^ currents by selective inactivation of axonal channels with a voltage pre-pulse. Their experiments started from a voltage clamp protocol without a pre-pulse that did not isolate Na^+^ currents. Our optimization started from a voltage trajectory with an initial level of *x*_min_ and increased to *x*_max_ over a duration of 158 ms, which did not permit APs to escape. The voltage profile generated at each iteration also did not activate spikes in the hybrid model. The final optimized trajectory by PSO increased nonlinearly from *x*_min_ to *x*_max_ over a duration of 46 ms. We added a statement to the fifth paragraph in the Discussion to compare the axonal responses evoked by two voltage-clamp protocols.

[1] Milescu LS, Bean BP, Smith JC. Isolation of somatic Na+ currents by selective inactivation of axonal channels with a voltage prepulse. J Neurosci. 2010 Jun; 30(22): 7740–8.
4. The optimal waveform was obtained here in the absence of an external stimulus, but would it be different if excitation were present?

Response: In our simulations, we designed the transmembrane voltage trajectory *V*_PSO_ to generate CSI of VGSCs in the absence of any external stimulus (i.e., in a quiescent node). This voltage trajectory was generated by applying PSO to maximize the fractions of Na_v_ 1.1 and Na_v_ 1.6 channels in the inactivated states while minimizing the fractions of the two VGSCs in open states. If activity was present in the node when generating the voltage profile *V*_PSO_, the initial states of the Na_v_ 1.1 and Na_v_ 1.6 channels would be markedly different than in the quiescent axon that we used, and this would likely result in a family of different *V*_PSO_, where each individual resulting *V*_PSO_ would depend not only on the membrane voltage *V*_m_(*t*) at the onset of *V*_PSO_, but on the time history of *V*_m_(*t*), as well.

However, and of paramount importance, we show that the *V*_PSO_ designed from the quiescent state remained effective at producing CSI and onset-free conduction block even in the presence of “intrinsic” activity generated by another external stimulus at a wide range of firing rates (Fig. 3).

5. The authors might want to emphasize/explain that the goal was not to block conduction in the entire axon by CSI, but only at one node (or a few).

Response: The objective of our study was to design a kilohertz frequency (KHF) current waveform to achieve conduction block without causing onset firing. We used an extracellular point source electrode placed over the central node to deliver PSO-based KHF currents to the model axon, which produced a locally-acting conduction block. The APs initiated at one end of the axon (i.e., simulated intrinsic activity) were unable to propagate to the node at the opposite end of the axon. That is, the nerve conduction was also blocked in the entire axon.

6. The study convinced me that, in general, a waveform can be designed to produce conduction block without onset response. The waveform discussed here works with a computational compartmental model that describes a certain type of axon, and the authors admit that it may not work with other types. Is there a strategy that would allow a clinical device to generate an optimal waveform for a specific patient? That is, without triggering an onset response during the optimization process, or at least reduce that. For example, wouldn't it be safe to just ramp the voltage up so very slowly, in hundreds of ms, say? Could you show a simulation with a much shallower ramp? If the optimization was started with a square waveform, I could see how the optimizer would stop when it was just shallow enough. Of course, I could be entirely wrong, and this waveform could really be the only one that works. Please explain and illustrate.

Response: Our engineering optimization approach with the PSO algorithm was developed to design KHF blocking waveforms using computational models, and although the optimization approach is not readily applicable to implementation in a clinical setting, the waveform that we designed could be implemented in practice using a voltage-to-current convertor or arbitrary waveform generator. If our model-specific optimized waveform designed here was not initially effective at producing onset-free conduction block in the experimental (clinical) setting, it could be the initial waveform for a subsequent optimization to alter the shape of the waveform to produce onset-free conduction block. However, the optimization process, at least as implemented here, requires a large number of iterations, and this may not be consistent with the limitations of a clinical setting.

Prior results suggest that onset-free conduction block cannot be achieved by simply ramping the voltage up very slowly, even in hundreds of ms. Miles *et al* [1] showed that ramped-amplitude waveforms did not eliminate the onset response in either the HH-type MRG model or in experiments on rat sciatic nerve. Their ramp durations ranged from 0 s to 60 s and still generated onset firing.

[1] Miles JD, Kilgore KL, Bhadra N, Lahowetz EA. Effects of ramped amplitude waveforms on the onset response of high-frequency mammalian nerve block. J Neural Eng. 2007 Dec; 4(4): 390–8.
7. Could you give estimates on how long would optimization take on a desktop computer?

Response: We used a desktop computer (8 cores/3.40 GHz) to estimate the time to complete the PSO run. When generating the voltage trajectory *V*_PSO_ for causing the CSI of VGSCs, the 21-node model was solved in the NEURON simulation environment (version 7.7) using a time step of *dt* = 0.005 ms and a simulation duration of *tstop* = 200 ms. In this case, completing 1000 generations took about 56.7 hours. When generating DC waveform to drive *V*_PSO_, we reduced the simulation duration in NEURON to *tstop* = 100 ms since *V*_PSO_ entered the plateau level over a duration of 46 ms. In this case, completing 1000 generations took about 96.1 hours.

Note the above estimations are dependent on the number of nodes in the model axons, the run parameters in NEURON (i.e., *dt* and *tstop*), the time step of the PSO algorithm, and, of course, the specifications of the computer processor.

**Reviewer #2**

**Abstract/Summary**

1. In previous papers, KHFAC is used instead of KHF.

Response: In previous papers, KHFAC was the acronym of kilohertz frequency alternating current. In our manuscript, KHF was the acronym of kilohertz frequency. We agree with the Reviewer and revised KHF to KHFAC.

2. Mention autonomic applications in both the abstract and summary

Response: Applications of conduction block in autonomic nerve fibers using KHFAC were added in the Abstract, Author summary, and Introduction.

**Introduction**

3. Has CSI been validated in different cell types? Several of the references are for cardiac cells. Include a list of what types of cells it has been observed in.

Response: Yes, closed-state inactivation (CSI) of voltage-gated sodium channels (VGSCs) has been validated in the cardiac cells [1-3], skeletal muscle [4], neuroblastoma [5, 6], squid giant axons [7], medullary raphe neurons [8], and dorsal root ganglion neurons [9, 10]. These references are included in the second paragraph of the Introduction in the revised manuscript.

[1] Dharmawan T, Nakajima T, Iizuka T, Tamura S, Matsui H, Kaneko Y, Kurabayashi M. Enhanced closed-state inactivation of mutant cardiac sodium channels (SCN5A N1541D and R1632C) through different mechanisms. J Mol Cell Cardiol. 2019 May; 130: 88–95.
[2] Scanley BE, Hanck DA, Chay T, Fozzard HA. Kinetic analysis of single sodium channels from canine cardiac Purkinje cells. J Gen Physiol. 1990 Mar; 95(3): 411–37.
[3] Lawrence JH, Yue DT, Rose WC, Marban E. Sodium channel inactivation from resting states guinea-pig ventricular myocytes. J Physiol. 1991 Nov; 443: 629–50.
[4] Groome J, Lehmann-Horn F, Holzherr B. Open- and closed-state fast inactivation in sodium channels: differential effects of a site-3 anemone toxin. Channels 2011 Jan-Feb; 5(1): 65–78.
[5] Aldrich RW, Corey DP, Stevens CF. A reinterpretation of mammalian sodium channel gating based on single channel recording. Nature. 1983 Dec 1–7; 306(5942): 436–41.
[6] Goldman L. Sodium channel inactivation from closed states: evidence for an intrinsic voltage dependency. Biophys J. 1995 Dec; 69(6): 2369–77.
[7] Armstrong CM. Na channel inactivation from open and closed states. Proc. Proc Natl Acad Sci U S A. 2006 Nov 21; 103(47): 17991–6.
[8] Milescu LS, Yamanishi T, Ptak K, Smith JC. Kinetic properties and functional dynamics of sodium channels during repetitive spiking in a slow pacemaker neuron. J Neurosci. 2010 Sep; 30(36): 12113–27.
[9] Cummins TR, Howe JR, Waxman SG. Slow closed-state inactivation: a novel mechanism underlying ramp currents in cells expressing the hNE/PN1 sodium channel. J Neurosci. 1998 Dec; 18(23): 9607–19.
[10] Herzog RI, Cummins TR, Ghassemi F, Dib-Hajj SD, Waxman SG. Distinct repriming and closed-state inactivation kinetics of Nav1.6 and Nav1.7 sodium channels in mouse spinal sensory neurons. J Physiol. 2003 Sep; 551(Pt 3): 741–50.
4. Mention that the Markov model is validated across electrophysiological data from all VGSC isoforms (Balbi article)

Response: We agree with the Reviewer and added this in the third paragraph of the Introduction in the revised manuscript.

5. There are a lot of different optimization techniques, why did you chose PSO?

Response: PSO is an effective search method for dealing with non-linear optimization problems by moving the particles to a “preferred” position in their constrained domains [1, 2]. It has been proven effective for determining sequences of real numbers in a non-linear system [2-4], which was well suited to designing our voltage and current waveforms. We added text to the third paragraph of the Introduction to make this clear.

[1] Kennedy J, Eberhart R. Particle swarm optimization. IEEE Int Conf Neural Networks Proc. 1995; 4: 1942–8.
[2] Kennedy J. Particle Swarm Optimization. Encyclopedia of Machine Learning. Springer, Boston, MA, 2011.
[3] Peña E, Zhang S, Patriat R, Aman JE, Vitek JL, Harel N, Johnson MD. Multi-objective particle swarm optimization for postoperative deep brain stimulation targeting of subthalamic nucleus pathways. J Neural Eng. 2018 Dec; 15(6): 066020.
[4] Peña E, Zhang S, Deyo S, Xiao Y, Johnson MD. Particle swarm optimization for programming deep brain stimulation arrays. J Neural Eng. 2017 Feb; 14(1): 016014.

**Results**

There are a couple of things that are fully explained in the methods, but could use a little bit more explanation here

6. State why were HH channels unable to represent the dependence of inactivation on activation.

Response: The HH models represent an ionic channel as an assembly of several independent gating variables, which are unable to represent the dependence of inactivation on activation. We added text to the first paragraph of the first subsection of the Results in the revised manuscript.

7. Could use a little more background on the PSO algorithm before launching into the way you are using it. Mention why it was selected.

Response: PSO is a search algorithm inspired by the social behavior of swarms. It finds the optimal or near-optimal solutions to numerical and qualitative problems by adjusting the trajectories of individual particles in the defined search space of an objective function, which is particularly applicable to solving our waveform optimization. We added text to the third paragraph of the Introduction to describe why PSO was selected.

8. “We defined a population of 50 particles as the membrane voltages used to control a series of voltage clamps at node 10.” Clarify that the membrane voltage is a voltage profile.

Response: The text was modified to make clear that the transmembrane voltage is a voltage profile.

These items don’t seem to be well explained in the methods either

9. Why only in the middle nodes?

Response: KHFAC waveforms are applied in practice using comparatively small electrodes and generate a locally-acting conduction block in peripheral nerves. In our simulations, there were 21 nodes in the model axon and the simulated block electrode was placed above the central node (i.e., node 10). We implemented Markov-type voltage-gated sodium channels (VGSCs) in node 6 to node 14, which were close to the block electrode. The other nodes far away from the site of stimulus delivery were modeled with HH-type VGSCs. As the Markov-type representations are substantially more computationally-demanding than HH-type representations, the hybrid model was used to reduce overall computation demands. We added text in the third subsection of the Methods to make this clear.

The specific number of Markov-type nodes did not alter the block threshold or lack of onset response in the hybrid model (see Fig S5), and these results were described in the fourth paragraph in Discussion.

10. Why change the conductance densities? Is there a reference for this?

Response: In the original MRG myelinated axon model, each node included fast Na^+^ current *I*_Naf_ and persistent Na^+^ current *I*_Nap_. *I*_Naf_ was responsible for AP initiation in each node (maximum conductance: $\bar{g}_{\mathrm{Naf}}=$ 3 S/cm^2^), and *I*_Nap_ was responsible for generating a non-inactivating current that produced the afterpotentials (maximum conductance: $\bar{g}_{\mathrm{Nap}}=$ 0.01 S/cm^2^). We substituted the HH-type models of *I*_Naf_ and *I*_Nap_ in node 6 to node 14 with Markov-type models of Na_v_ 1.1 and Na_v_ 1.6 channels, respectively. We modified only the maximum conductance of *I*_Nav11_ and did not change any parameter of their transition rates between consecutive states. The maximum conductance $\bar{g}_{Nav11}$ = 3 S/cm^2^ of Na_v_ 1.1 channels was too small to propagate faithfully an AP from node 0 to node 20 (Fig. R2). Therefore, we set $\bar{g}_{Nav11}$ to the minimal value (11.9 S/cm^2^) to propagate faithfully spike trains at rates of up to 400 spikes per second. At $\bar{g}_{Nav11}=$ 11.9 S/cm^2^, there was no attenuation in the APs as they propagated along the axon (please see Fig S1). This is described in the third subsection of the Methods. The maximum conductance of Na_v_ 1.6 channels was set to $\bar{g}_{Nav16}=$0.01 S/cm^2^, which was identical to that of *I*_Nap_.

**Fig. R2. Propagation of a test AP initiated in node 0 along the 21-node and 10 μm model.** A single test pulse (width: 0.1 ms and amplitude: 2.5*I*_th_) was delivered at the node 0. Markov-type *I*_Nav11_ and *I*_Nav16_ were implemented in node 6 to node 14. The maximum conductance of *I*_Nav11_ was $\bar{g}_{Nav11}=$3.0 S/cm^2^, and the maximum conductance of *I*_Nav16_ was $\bar{g}_{Nav16}=$0.01 S/cm^2^.

KHFAC waveform

11. Describe why you thought this would work. KHFAC generates a dynamic depolarization which should be able to drive the membrane voltage?

Response: The KHFAC generated a dynamic depolarization in the nodes close to the block electrode (see Fig. 4A). The intrinsic low-pass filtering and rectification of a.c. electrical signals by cell membrane [1, 2] prevented neural firing from following the KHFAC stimulus. The envelop of the KHFAC waveform drove the Na_v_ 1.1 channels to CSI (see Fig. 4B), which produced conduction block without causing onset firing. This is described in the second paragraph of the third subsection of the Results (page 10) in the revised manuscript.

[1] Hutcheon B, Yarom Y. Resonance, oscillation and the intrinsic frequency preferences of neurons. Trends Neurosci. 2000 May; 23(5): 216–22.
[2] Grossman N, Bono D, Dedic N, Kodandaramaiah SB, Rudenko A, Suk HJ, et al. Noninvasive deep brain stimulation via temporally interfering electric fields. Cell. 2017 Jun; 169(6): 1029–1041.
12. What is the purpose of the 1.5 scale factor? If you have created an optimized waveform, why are you changing it?

Response: After designing a transmembrane voltage trajectory that moved the two types of VGSCs into CSI, we applied PSO to determine the stimulation waveform *I*_PSO_ required to drive that voltage trajectory in the 21-node axon. The resulting DC waveform *I*_PSO_ was not charge-balanced, and thus could cause irreversible faradaic reactions at the electrode - electrolyte interface [1], which may result in electrode and or nerve damage when applied for long periods of time [2-4]. We therefore used a scaled version of the DC waveform as the envelope of symmetric rectangular charge-balanced biphasic pulses, applied at 10 kHz to block nerve conduction. However, there was a time delay between the onset of resulting KHFAC waveform and complete conduction block, which depended on the scale factor (see Fig. 5B). A scale factor of 1.5 enabled the PSO-based KHFAC waveform to generate conduction block after a minimum time delay, i.e., 48 ms. This is described in the first paragraph of the third subsection of the Results in the revised manuscript.

[1] McCreery DB, Agnew WF, Yuen TG, Bullara LA. Comparison of neural damage induced by electrical stimulation with faradaic and capacitor electrodes. Ann Biomed Eng. 1988; 16(5): 463–81.
[2] Franke M, Vrabec T, Wainright J, Bhadra N, Bhadra N, Kilgore K. Combined KHFAC+DC nerve block without onset or reduced nerve conductivity after block. J Neural Eng. 2014 Oct; 11(5): 056012.
[3] Bhadra N, Kilgore KL. Direct current electrical conduction block of peripheral nerve. IEEE Trans Neural Syst Rehabil Eng. 2004 Sep; 12(3): 313–24.
[4] Cogan SF. Neural stimulation and recording electrodes. Annu Rev Biomed Eng. 2008; 10: 275–309.

**Discussion**

13. I don’t think that applying a waveform that has been optimized to one model to another model produces a valid comparison. I not sure what you are trying to demonstrate by altering the number of Markov-type nodes? It isn’t clear at this point why you only changed a select number of nodes.

Response: These sensitivity analyses were conducted to assess the robustness of the resulting optimized waveform. In *in vivo* experiments, a KHFAC waveform is commonly delivered using an extracellular electrode and can affect a number of axons with different fiber diameters and different electrode-to-fiber distances. Our KHFAC waveform was designed in a 21-node model at a specific diameter of 10 μm and a specific electrode-fiber distance of 1 mm. The block threshold is dependent on electrode-fiber distance and fiber diameter [1-3]. Therefore, we examined whether our PSO-based KHFAC waveform optimized in a specific model produced onset-free conduction block in another model with different diameters and at other locations. We added text in the second paragraph of Discussion in the revised manuscript to make this clear.

As described above (please see response to Comment 9), we implemented Na_v_ 1.1 and Na_v_ 1.6 channels only in the central nine nodes of a 21-node model to reduce overall computational demands. To examine how the number of Markov-type nodes affected our simulations, we implemented Na_v_ 1.1 and Na_v_ 1.6 channels in different number of nodes and re-examined the conduction block by using PSO-based KHFAC waveform. The specific number of Markov-type nodes did not alter the block threshold or the lack of an onset response in the hybrid model (see Fig S5), and therefore our use of a model with only 9 nodes represented with Markov kinetics was justified. We added text in the fourth paragraph of the Discussion to make this clear.

[1] Ackermann, D. M. Jr., Bhadra, N., Foldes, E. L., Wang, X. F. & Kilgore, K. L. Effect of nerve cuff electrode geometry on onset response firing in high-frequency nerve conduction block. IEEE Trans Neural Syst Rehabil Eng. 2010 Dec; 18(6): 658–65.
[2] Pelot NA, Behrend CE, Grill WM. Modeling the response of small myelinated axons in a compound nerve to kilohertz frequency signals. J Neural Eng. 2017 Aug; 14(4): 046022.
[3] Bhadra N, Lahowetz EA, Foldes ST, Kilgore KL. Simulation of high-frequency sinusoidal electrical block of mammalian myelinated axons. J Comput Neurosci. 2007 Jun; 22(3): 313–26.
14. In reference to the Gerges paper: “The amplitude transition in their KHF waveform was opposite with our PSO based waveform” This may be true, but they are also changing the frequency. Comparing your results with the Miles paper is a more appropriate comparison.

Response: We agree with the Reviewer, and deleted the sentence that compared our results with the paper by Gerges *et al*.

15. This is basically an KHFAC amplitude ramping waveform that has been tested in vivo. Spend more time talking about the Miles paper and include this reference as well

Vrabec, T.L., et al., Reduction of the onset response in kilohertz frequency alternating current nerve block with amplitude ramps from non-zero amplitudes. Journal of neuroengineering and rehabilitation, 2019. 16(1): p. 80-80.

Response: Miles *et al* showed that ramping KHFAC waveforms from zero amplitude to block threshold did not eliminate the onset response in either the MRG model or in experiments on rat sciatic nerve. Vrabec *et al* reported that applying a subthreshold KHFAC (50, 75, and 90% of block threshold) for 15 s and then ramping up to 125 % of block threshold was successful in eliminating the onset response in rat sciatic nerve. These references are described in the sixth paragraph of the Discussion in the revised manuscript.

16. The Franke paper included high capacitance materials as well as a charge balanced waveform.

Response: We added text to the revised manuscript to make this clear.

17. Mention the ramping papers again in the paragraph about the bipolar electrodes. The ramping papers both used bipolars and are a direct comparison to what you are doing.

Response: The ramping paper by Miles *et al* used the tripolar electrode, and the ramping paper by Vrabec *et al* used the bipolar electrode. Therefore, we compared our simulation results using bipolar cuff with the paper by Vrabec *et al*, and compared our simulations of using tripolar electrode with the reference by Miles *et al*. This is described in the fifth paragraph of the Discussion in the revised manuscript.

**Methods**

Implementation of Markov type VGSCs

18. There still isn’t any reason for only doing nodes 6-14

Response: We added text in this section to explain why we only implemented Markov-type VGSCs in nodes 6-14 (to reduce overall computational demands), and the detailed explanation is in the response to Comment 9, above.

19. It looks like the reason that you needed to change the conductance was because of the transition between HH and Markov channels which begs the question of why all the nodes aren’t Markov?

Response: The maximum conductance $\bar{g}_{Nav11}=$ 3 S/cm^2^ of Na_v_ 1.1 channels was too small to faithfully propagate an AP from node 0 to node 20 (Fig. R2). Therefore, we set $\bar{g}_{Nav11}$ to the minimal value (11.9 S/cm^2^) to propagate faithfully spike trains at rates of up to 400 spikes per second. At $\bar{g}_{Nav11}=$ 11.9 S/cm^2^, there was no attenuation in the APs as they propagated along the axon (see Fig S1).

The specific number of Markov-type nodes did not alter the block threshold or absence of onset response in the hybrid model (see Fig S5). That is, the results of implementing Markov-type VGSCs in all the nodes were the same with those of only doing nodes 6-14, while the latter produced a substantial reduction in computational demands.

Particle Swarm Optimization

20. Why was this optimization approach chosen? (or include this in the results section)

Response: Please see responses to Comments 5 and 7.
